# Prevalence of Intestinal Parasitosis in Guinea: Systematic Review of the Literature and Meta-Analysis

**DOI:** 10.3390/pathogens12020336

**Published:** 2023-02-16

**Authors:** Timothé Guilavogui, Stéphane Verdun, Akoï Koïvogui, Eric Viscogliosi, Gabriela Certad

**Affiliations:** 1 Centre National de la Recherche Scientifique, Institut National de la Santé et de la Recherche Médicale, Centre Hospitalier Universitaire de Lille, Institut Pasteur de Lille, U1019-UMR 9017-Centre d’Infection et d’Immunité de Lille, Université de Lille, 59000 Lille, France; 2 Unité d’Appui à la Gestion et la Coordination des Programmes, Ministère de la Santé, Conakry 585, Guinea; 3 Délégation à la Recherche Clinique et à l’Innovation, Groupement des Hôpitaux de l’Institut Catholique de Lille, 59000 Lille, France; 4 Comité Départemental des Cancers (CDC-93), CRCDC-IDF, Site de Seine-Saint-Denis, 93146 Bondy, France

**Keywords:** intestinal parasitosis, meta-analysis, systematic review, prevalence, Guinea

## Abstract

Background: Intestinal parasitosis constitute a major public health issue, particularly in sub-tropical and tropical areas. Even though they are classified as neglected tropical diseases, no national study has been carried out recently in Guinea to estimate the prevalence of intestinal parasitosis. Objective: A systematic review and meta-analysis aimed to determine the overall prevalence of intestinal parasitosis in Guinea. Method: The PRISMA method was used to perform a systematic review and meta-analysis. The studies carried out in order to study intestinal parasitosis in Guinea and published between 2010 and 2020 were searched in online public databases. The prevalence of parasitosis was calculated by a random-effects meta-analysis. Subgroup comparisons were performed using Q-tests. Statistical analyses were performed with the R software. This review was registered with PROSPERO under the identification number CRD42022349743. Results: 69 studies were selected out of 1230 studies identified in online public databases. The meta-analysis involved 44,186 people with an overall prevalence of intestinal parasitic infections of 52%. Conclusions: This is the first study in Guinea to assess the prevalence of intestinal parasitic infections in different regions of the country. It was found that intestinal parasitosis are a real health problem in Guinea, hence, the need to put in place national strategies for regular control.

## 1. Introduction 

Intestinal parasitic infections are widespread in the world, with school-aged children being the most affected, with prevalences in some geographic areas of more than 50% [1]. According to the World Health Organization (WHO), in the world, more than 1.5 billion people are infected by intestinal parasites, and 450 million are seriously ill, with an estimated mortality rate of 155,000 cases per year [2]. The promiscuity and lack of hygiene associated with poverty favor their expansion. In some regions of the world, particularly in sub-tropical and tropical areas, intestinal parasitic infections are endemic and remain one of the leading causes of increased morbidity and mortality, justifying the mobilization of resources and actions aimed at their control and eradication [3,4].

Developing countries are the most affected by intestinal parasitic infections, particularly in areas such as sub-Saharan Africa, South and Central America, China, and East Asia [1,5]. This situation constitutes an obstacle to socio-economic development [6]. Intestinal parasitic infections are caused by both helminths and protozoans. Soil-transmitted helminths include, among others, *Ascaris lumbricoides,* responsible for ascariasis; *Ancylostoma duodenale,* responsible for hookworm infection; and *Trichuris trichiura,* responsible for trichocephalosis [5]. Among the water-transmitted helminths, *Schistosoma mansoni*, responsible for schistosomiasis, is one of the most important blood flukes in Africa [6,7]. The main modes of contamination by these parasites are the eggs present in the stools of infected people or the larvae. Adult worms, which are located in the gut of an infected person, can produce thousands of eggs every day, contaminating environments that lack adequate sanitation [8,9].

Among the most important intestinal protozoans are *Entamoeba histolytica*, *Giardia intestinalis*, *Blastocystis,* and *Cryptosporidium* spp. [10]. The latter, in some cases, can be the cause of serious gastrointestinal infections resulting in high morbidity and mortality, particularly in children and immunocompromised patients [11].

Despite their health impact, these parasites remain neglected by health authorities, even though most of the diseases they cause have been categorized as neglected tropical diseases (NTDs) [1,2].

In Africa, it is difficult to have reliable epidemiological data concerning parasitic intestinal infections due mainly to the underreporting of cases. However, some studies describe a high prevalence of intestinal parasitosis, even if this prevalence is variable from one region to another. For instance, 84.7% of intestinal parasitosis have been reported in Burkina Faso [12], 15.8% in Senegal [13], and 55.2% in Côte d’Ivoire [14]. It is important to mention that in sub-Saharan countries such as Ethiopia, a prevalence of intestinal parasitic infection greater than or equal to 50% is classified as high [15]. Particularly, in Guinea, according to the prevalence of geo-helminthiasis, the health districts have been classified as hyper-endemic (50% of prevalence or more), meso-endemic (20–49% of prevalence), and hypo-endemic (less than 20% of prevalence) [16].

A national survey performed in Guinea in 1995 reported a 70% prevalence of geo-helminthiasis in school-aged children in the four natural regions of the country: Lower Guinea, Middle Guinea, Upper Guinea, and Forest Guinea [4]. Nevertheless, to our knowledge, no national study has been carried out to estimate the prevalence of intestinal parasitosis or even to assess its impact on the health of the population since then.

Guinea launched a national health policy (NHP) in 1996 [17]. Dysfunctions in the system were noticed, and the Ministry of Health organized a review of the health system in 2000. This review recommended the development of the new NHP together with a National Health Development Plan (NHDP) by 2010 [16]. In order to eradicate intestinal parasitosis in Guinea, the National Program to Control Neglected Tropical Diseases (NPCNTD) was set up in 2010 as part of the first NHDP, before being attached to the National Directorate of Major Endemics and Illness Control in 2018. To intensify the fight against NTDs initiated in 2010, this NPCNTD implemented several community actions. Efforts were mainly focused on the control of chemo-preventive diseases (onchocerciasis, lymphatic filariasis, trachoma, schistosomiasis, and geo-helminthiasis) and those requiring curative treatment (leprosy, Human African Trypanosomiasis, and Buruli ulcer). Specifically, since 2014, mass treatment for geo-helminthiasis has been integrated with treatment for lymphatic filariasis and onchocerciasis. Then the health districts endemic to geo-helminthiasis benefited progressively from albendazole treatments for populations aged 5 years and older [16] (Table 1). Parasitosis due to protists such as *Cryptosporidium* was not included in the fight against NTDs. However, in 2017, according to the health statistics yearbook of the Ministry of Health, the annual incidence rate of intestinal helminthiasis was estimated at 26 per 1000 habitants, ranking it behind malaria and respiratory infections [16].

In the absence of recent national epidemiological studies in the field evaluating the evolution of the prevalence of intestinal parasitosis in the country, it seems necessary to perform a situational analysis before planning new studies and strategies to control NTDs, in particular intestinal parasitosis. Thus, the aim of this study was to undertake a systematic review and meta-analysis to evaluate and describe the research estimating the prevalence of intestinal parasitosis in Guinea in order to synthesize the data, describe the evolution of the prevalence, and identify the existing gaps as well as future research priorities.

## 2. Methods

### 2.1. Documentary Search Strategy

A systematic review of the literature and a meta-analysis were carried out using the Preferred Reporting Items for Systematic Review and Meta-Analysis (PRISMA) method [18]. We searched in the PubMed/Medline, Google Scholar, and ResearchGate databases (for peer-reviewed journal articles) and in the Library of Gamal Abdel Nasser University (UGAN) in Conakry (for dissertations or theses of graduate students in the departments of medicine, pharmacy, and biomedical sciences) to compile all the studies carried out on intestinal parasitosis in Guinea and published between 2010 and 2020. Two reasons motivated the choice of this period: (i) Theses prior to 2010 were not accessible in the database of the Library of Gamal Abdel Nasser University in Conakry, and although the search was done in a digital catalog, only the papers formats of these dissertations were accessible in the library of the UGAN.; (ii) The National NTD Control Program, which defines national strategic guidelines, began after 2010.

For the documentary search in each database, the keyword pair “Guinea” and “Intestinal parasitosis” was initially used. Then, in a second step, the keyword “Guinea” was used in association with each intestinal parasite or parasitic infection (i.e., “Guinea & *A. duodenale*” or “Guinea & Ancylostomiasis”…). The exhaustive list of the parasites sought was: Hookworms (*A. duodenale* and *Necator americanus*), Eel (*Strongyloides stercoralis)*, *A. lumbricoides*, Whipworm (*T. trichiura*), Pinworm (*E. vermicularis*), Trichina (*Trichinella spiralis*), *S. mansoni* (*S. mansoni, S.intercalatum, S.japonicum and S.mekongi*), Tapeworms (*Taenia saginata,* and *Taenia-solium, Hymenolepis diminuta*, *Hymenolepis nana*), *Trichomonas intestinalis*, *Balantidium coli*, *Cryptosporidium*, *Cryptosporidium hominis*, *Cryptosporidium parvum*, *Blastocystis, Cystoisospora belli, Isospora belli*, *Entamoeba histolytica*, *Fasciolopsis buski, G. intestinalis*, (formerly called *Giardia lamblia, Giardia duodenalis,* and *Lamblia duodenalis)*.

### 2.2. Study Selection Criteria

Criteria were established for eligibility before beginning the search, as follows:

Inclusion criteria:

- Language of publication/written: English or French
- Year of publication/report: from 2010 until 2020
- Study design: observational studies (cross-sectional, case-control, cohort).
- Outcome: prevalence of intestinal parasites and/or associated factors
- Study population: not restriction
- Study setting: at institution or community based
- Study country: Guinea
- Diagnostic modality: stool examination
- Type of parasite: either protozoa or helminths or both
- Types of articles: both published and unpublished including dissertations or theses accessible in the database of the UGAN in Conakry.
- Types of publication: peer-reviewed full-text articles.
- Exclusion criteria:
- Publications dating before 2010
- Publications reporting prevalence after antiparasitic treatment.
- Duplicates articles
- Dissertations of graduate students deposited in university libraries in Conakry before 2010
- Articles and dissertations on intestinal parasitosis carried out outside Guinea
- Articles that failed to report the number of study participants and number of cases

### 2.3. Search Strategy

The articles and theses/dissertations selected were read and analyzed in accordance with a reading grid, and the extracted data included: (i) name of authors; (ii) diploma obtained as a result of the study in the case of theses/dissertations (MD: medical doctor; PharmD: pharmacy doctor; master or MSc: master in sciences), and the reference number of the study; (iii) year of publication (year of publication by the editor or year of submission of the thesis to the university library); (iv) year of observation (period of beginning of inclusions or selection of the study population); (v) age range of the study population (according to age, the study population was divided into 5 groups: children between 0 and 5 years old, children between 0 and 17 years old, adults, mixed population (children and adults), and pregnant women); (vi) study setting; (vii) study site (area corresponding to the place of residence of the population included in the study. For the analysis of the study site, the districts were grouped by natural regions (Conakry, Lower Guinea, Middle Guinea, Upper Guinea, and Forest Guinea). Conakry was distinguished from Lower Guinea to highlight its cosmopolitan character and the fact that it was the only city in the country with an institute of medicine and pharmacy, since independence until 2006. Lower Guinea (20% of the area), is a maritime region characterized by high rainfall; Middle Guinea (18%) is a region of mountains and many rivers, Upper Guinea (22%) is the driest region in the country, Forest Guinea (40%) has a humid subtropical climate [16]; (viii) type of laboratory techniques: standard direct microscopic examination, direct microscopic examination after special stains and the concentration or staining/discoloration techniques performed (formol-ether, Kato–Katz, Willis, Ziehl –Neelsen) were listed; (ix) overall prevalence (any parasite); (x) type of parasites and the number of cases observed.

The prevalence of each of the most frequent parasitosis in the literature was retrieved over three periods: before 2010 (Period 1 (P1)), between 2010 and 2013 (Period 2 (P2)), and from 2014 to 2020 (Period 3 (P3)). These periods were chosen considering the application of the NHDP policy (Table 1).

### 2.4. Data Extraction

Studies were screened independently by two authors, first based on titles and abstracts. The full texts of papers identified as being potentially relevant for inclusion were retrieved and independently assessed by the same collaborators. Data extraction format for the pooled prevalence was facilitated using a Microsoft Excel sheet where data pertaining to the authors’ names, publication year, study design, study setting, study area, region, technique of stool examination, sample size, and prevalence of intestinal parasitic infection were listed.

### 2.5. Statistical Analysis

The prevalence of parasitosis was calculated by a random-effects meta-analysis (heterogeneity between studies was expected). A logistic regression model with random intercept was used to estimate prevalence, with logit transformation. Confidence intervals of the estimates for each study were calculated with the Clopper–Pearson method (exact binomial interval). Subgroup comparisons were performed using Q-tests. The general significance level was set at a *p*-value below 0.05. Statistical analyses were performed with the R software version 4.0.5 [19]. The R code used for the analysis and the database were added as Supplementary File S1.

### 2.6. Protocols and Registration

This systematic review and meta-analysis were registered on PROSPERO under the registration number CRD42022349743 and can be accessed at https://www.crd.york.ac.uk/PROSPERO (accessed on 28 July 2022).

## 3. Results

Characteristics of the Included Articles

**Figure 1:**
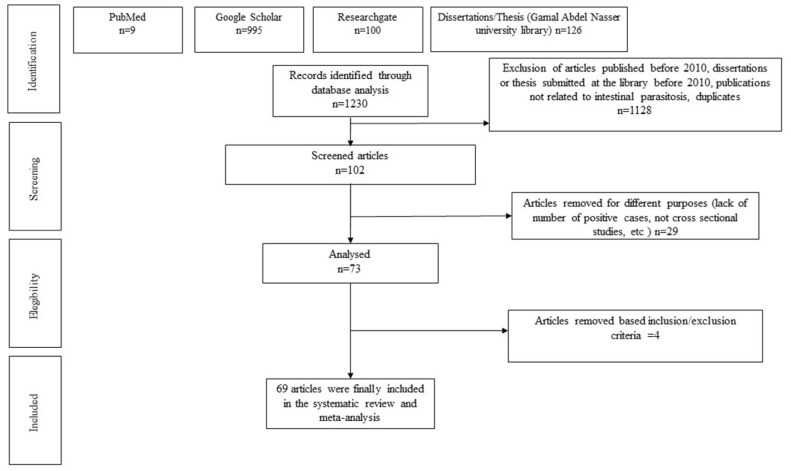
PRISMA chart flow showing article selection process.

A total of 1230 studies were initially identified in the queried databases. Overall, 1128 studies were excluded during the first selection phase according to the following reasons: exclusion of articles published before 2010, dissertations or theses submitted at the library before 2010, publications not related to intestinal parasitosis, and duplicates. Out of the 102 publications or documents, after a second selection phase, 69 studies met the study criteria (Figure 1).

In total, 100% of selected studies were cross-sectional, and 68% described the frequency of intestinal parasitosis in people consulting a health care structure; 32% were performed in institutions such as schools, orphanages, universities, or jails. Parasitological examination techniques were not described in 10 studies. In the remaining 59 studies, a microscopic examination of fresh stools was performed, as this is the most commonly used laboratory test for stool sample analysis. This standard examination was followed by a modified Ziehl–Neelsen staining technique in only two studies or by the Kato–Katz technique in ten studies (Table 2).

The studies were carried out in the natural regions of Guinea as follows: 34 in Conakry, 8 in Lower Guinea, 5 in Middle Guinea, 4 in Upper Guinea, 16 in Forest Guinea, and 1 multicentric study in Conakry and Lower Guinea. Twelve studies included children aged 0–5 years; twenty studies included children of less than 18 years old; twenty-seven included a mixed population (children and adults); and ten included only pregnant women (Table 2). Ten studies were carried out in P1, seventeen in P2, and forty-two in P3 (Table 2).

## 4. Pooled Prevalence of Intestinal Parasitic Infections in Guinea

**Figure 2:**
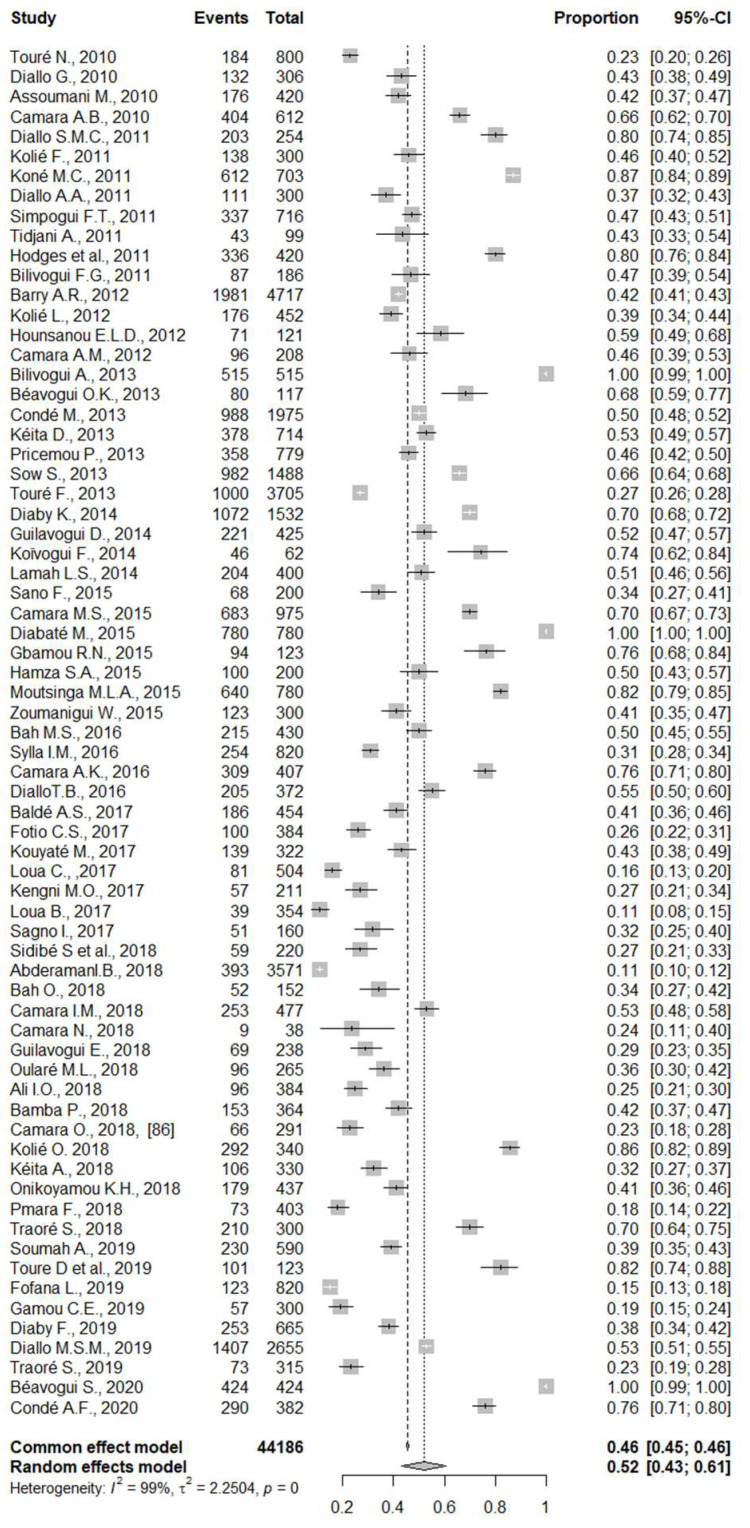
Forest plot representing the pooled prevalence of intestinal parasitic infections in Guinea.

The meta-analysis involved 44,186 people with an overall prevalence of intestinal parasitic infections of 52% (95 % CI: 43, 61) (Figure 2). A substantial heterogeneity was observed in the estimation of this pooled prevalence (I2 = 99%, *p* = 0).

## 5. Subgroup Analysis

### 5.1. Analysis by Type of Intestinal Parasitic Infections

**Figure 3:**
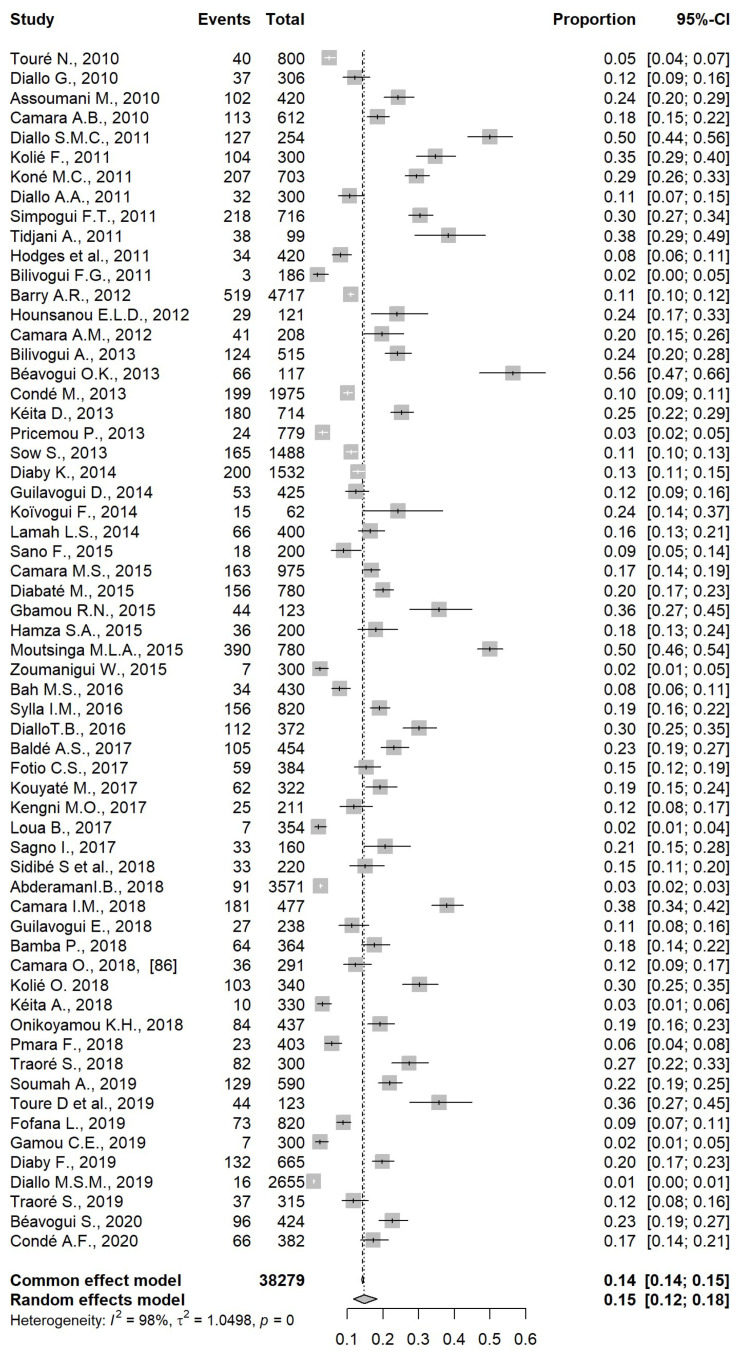
Forest plot representing the pooled prevalence of infections by Ascaris lumbricoides in Guinea.

We performed subgroup analysis by types of intestinal parasitic infections. Overall, helminths were predominant in comparison with protozoans with *A. Lumbricoides* (Figure 3), hookworm, and *S. mansoni* being the helminths most frequently found, with a prevalence of 15% (95% CI: 12,18), 6% (95% CI: 5,8), and 5% (95% CI: 3,7), respectively. Among protozoans, *E. histolytica* was predominant with a prevalence of 4% (95% CI: 3,5) (Table 3). The pooled prevalence of soil-transmitted helminth infections was 12%.

### 5.2. Analysis by Type of Population

**Figure 4:**
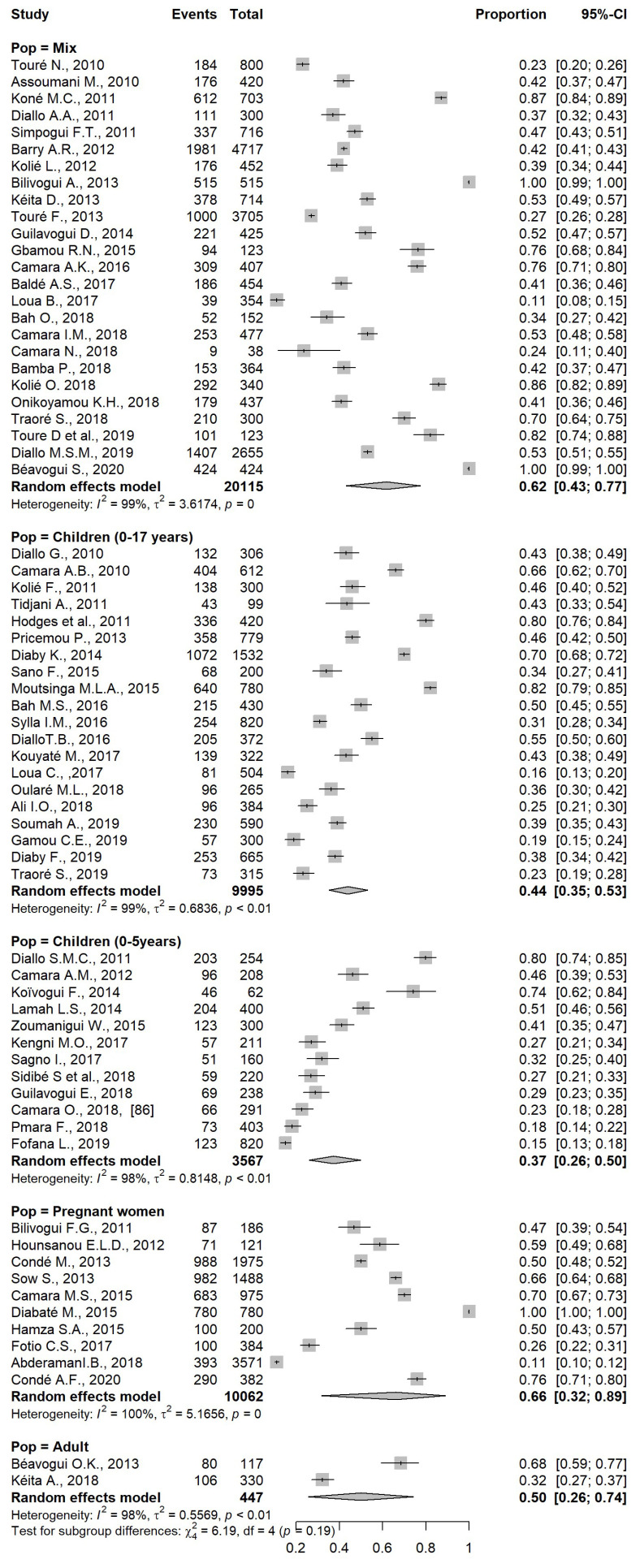
Forest plot of subgroup analysis based on types of population.

According to the type of study, the population was divided into 5 groups: children from 0 to 17 years, children from 0 to 5 years, adults, mixed population (children and adults), and pregnant women (without taking into consideration the age). The overall prevalence of intestinal parasites among pregnant women was 66 % (95% CI: 32, 89), the group with the highest prevalence, followed by 62% of prevalence in the mixed population. The young children were less infected, with a prevalence of 37 % (95 % CI: 26, 50) when compared to adults (Figure 4). The differences in prevalence between these subgroups were not statistically significant. A. lumbricoides was the parasite more frequently found in pregnant women (11%) and children (17%). S. mansoni was the parasite most frequently found in adults, with a prevalence of 14% (95 % CI: 11, 18).

### 5.3. Analysis by Study Setting

**Figure 5:**
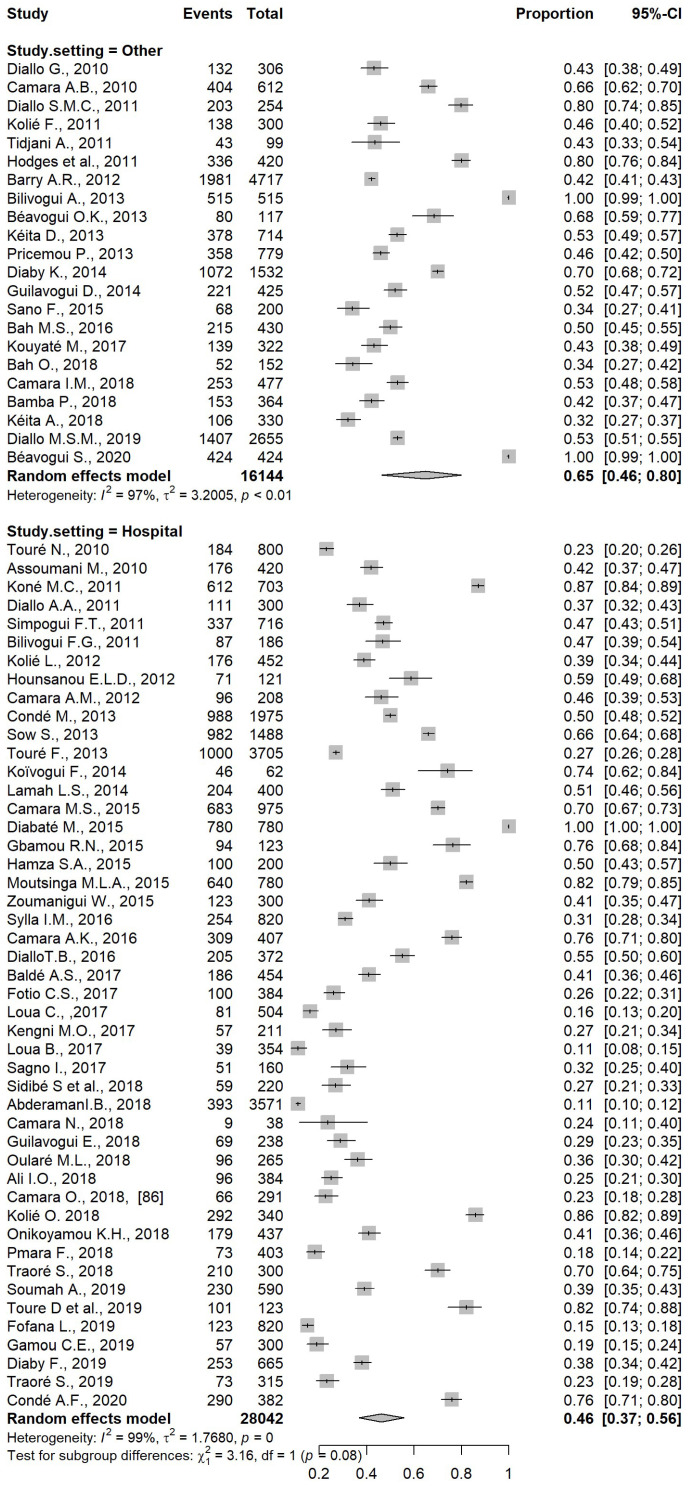
Forest plot of subgroup analysis based on study setting.

Subgroup analysis by setting showed a higher prevalence in studies conducted in the community than in those studies performed at health care centers, with a pooled prevalence of 65% (95% CI: 46, 80) and 46% (95% CI: 37, 56), respectively. However, this difference was not significant (Figure 5).

### 5.4. Analysis by Region

There was no statistically significant difference in parasite prevalence according to the study region. However, when the association between the regions and the types of parasites was determined, the geographic occurrence of all types of parasites differed within the five regions, with the exception of S. stercoralis. Upper Guinea was the region with the highest prevalence of A. duodenale, Trichine, and H. nana. In addition, Upper Guinea together with Forest Guinea were the regions with the highest prevalence of S. mansoni. Middle Guinea had the highest prevalence of tapeworms. The prevalence of A. lumbricoides was higher in Conakry and Middle Guinea (Table 4).

### 5.5. Analysis by Period

**Figure 6:**
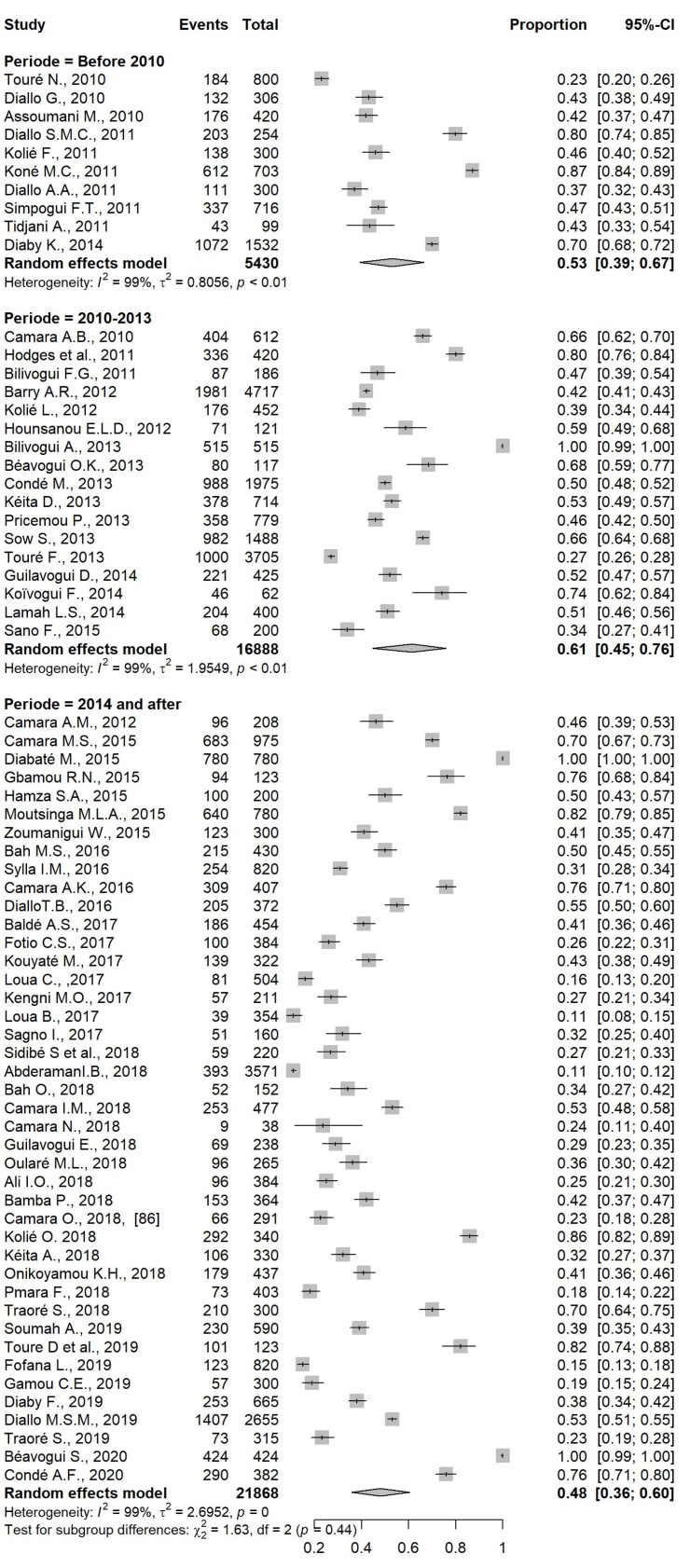
Forest plot of subgroup analysis based on study period.

Subgroup analysis of the proportions included in all selected studies showed a prevalence of intestinal parasitic infections as follows: 53% for P1 (before 2010), 61% for P2 (between 2010 and 2013), and 48% for P3 (2014 and after). This difference was not statistically significant. On the other hand, in Upper Guinea, Forest Guinea, and Conakry, a higher prevalence (all parasitosis combined) was noticed before 2014, but a significant decrease in prevalence during P3 was observed in all these localities (*p* < 0.01) (Figure 6).

### 5.6. Other Parasites

Other parasites were detected, but the studies reporting them were very few, and they could not be included in the meta-analysis. Overall, 13 cases of *H. diminuta* were reported in 1 study (rank: 53), 51 cases *of F. buski* in 2 (rank: 20, 56)*,* 67 *C. parvum* cases, and 1 case of *Isospora* infection were reported in 2 studies (rank: 31, 62), among immunodepressed patients, 12 cases of *Balantidium* were reported in three studies (rank: 40, 46, and 67).

## 6. Discussion

The present systematic review and meta-analysis assessed the prevalence of intestinal parasite infections in the general population of the country, establishing an overall prevalence of 52%. To our knowledge, this is the first study to provide a recent estimate of the prevalence of intestinal parasites in this country.

Before 2010, the prevalence of intestinal parasitosis in Guinea, according to the literature, was over 50% [4,88,89]. In this meta-analysis, this prevalence appears to vary from one study to another, sometimes over the same study period. However, even if a decrease in prevalence was observed here in P3 when compared to P1 or P2, prevalence remained high in all localities of the country. This slight decrease could be explained by the intensification of mass campaigns for the distribution of antiparasitic drugs in these localities [16]. However, the calculation of prevalence for P1 is based only on a limited number of studies that were available according to inclusion criteria and probably this is underestimating and not reflecting the real prevalence. In addition, it is well known that during the 2014–2016 Ebola virus disease (EVD) outbreak, routine health activities slowed down, including community-based activities such as deworming campaigns [3].

Data from this meta-analysis highlight a high rate of helminth infection, with the most prevalent being *A. lumbricoides* (15%)*, A. duodenale* (6.0%), and *S. mansoni* (5%).

Transmission of *A. lumbricoides* is oro-faecal, so the predominance of the parasite could be attributed to its high rate of reinfection when compared to other soil-transmitted helminths [90]. Additionally, its eggs can remain dormant and tolerate adverse conditions in the soil for up to 10 years [91]. Hookworm infection is usually associated with bare skin contact with contaminated soil containing third-stage larvae [9]. *S. mansoni* infection is a consequence of contact with environmental freshwater infested with parasite larvae. Then, the disease is especially prevalent in communities lacking access to piped drinking water and adequate sanitation [92]. Particularly, *S. mansoni* infection was more prevalent in Forest Guinea. This is consistent with the report of the Health Minister of Guinea (2010), which indicated that this zone was the most affected by this infection between 2000 and 2005 [16].

In other African countries, we found different results, with prevalences above 40% for certain parasites. As an example, in Mozambique, according to Augusto et al. [93], the parasites more prevalent were: *A. lumbricoides* (65.8%), *T. trichiura* (54.0%), hookworms (38.7%), *Entamoeba* spp. (31.2%), *G. intestinalis* (19.0%), *Taenia spp.* (5.8%), and *H. nana* (5.2%). In Ivory Coast, the study by Adoubryn et al. (14) showed a high prevalence of helminthes (*S. mansoni*: 35.5%, *N. americanus*: 25.9%, and *A. lumbricoides*: 5.2%). Concerning *A. lumbricoides*, the results are in agreement with another study reporting prevalences of 18% in South Africa [94] and South Asia [95], respectively, and 16% in Ethiopia [96].

In the current study, the pooled prevalence of soil-transmitted helminth infections was 12%. This prevalence was less than the ones reported in Rwanda (25.4%), Cameron (24%), Nigeria (54.8%), and Ethiopia (37%) [97].

The variation between regions and countries may be due to environmental factors such as temperature, humidity, rainfall, etc. In addition, differences in levels of sanitation should also be considered [97].

In addition, this study describes the prevalence of protozoans infecting the digestive tract such as *E. histolytica* and *G. intestinalis*. Concerning *Cryptosporidium*, this parasite was detected in 33% of patients infected with HIV, making it the most frequent parasitosis after ascariasis in this population. However, the presence of *Cryptosporidium* was not searched systematically since only two studies reported Ziehl–Neelsen staining as a specific diagnostic test [50,80].

In terms of the age of the population, the studies selected in this systematic review covered all age groups. Herein, the prevalence of intestinal parasites found in children less than 17 years old was 44%. When considering children under 5, the prevalence of intestinal parasites was 37%. This prevalence of intestinal parasites among under-five children was higher than that found in Saudi Arabia (17.7%) or Zambia (19.6%), lower than findings in Pakistan (52.8%), and slightly similar to the prevalence in Sudan (30%) [97,98]. Similar results were reported recently in Colombia, where prevalences of 37% (95% CI: 26–49) and 66% (95% CI: 52–78) were reported in preschoolers and schoolchildren [99]. Behavioral and social practices in children and their weak immune status, especially in under five, may account for the high frequency of intestinal parasitic infections [2,3,8]. With respect to pinworm prevalence, this was very low in children, in contrast with studies reporting this parasite as one of the most important intestinal pathogens in this group [100]. This could be explained by the fact that the identification of pinworms was performed only by microscopic examination of feces and not by the Scotch tape test, which is considered the gold standard for the diagnosis of *E. vermicularis* [101]. On the other hand, a prevalence of 66% of parasitic infections was found in pregnant women. This finding is similar to a prevalence of 66.7% reported in Burkina Faso [102]. In contrast, lower prevalences have been reported in Ghana (14.3%), Kenya (13.8%), or Ethiopia (27.32%) [103,104,105]. *A. duodenale* was among the most frequent type of parasite in this group. Infection by this parasite can lead pregnant women to severe anemia, and then there is an increased risk of morbidity and mortality for the mother and the baby after hookworm infections [105]. Maybe the calculation of prevalence for children and pregnant women was subject to bias considering that the detection of parasites is done systematically in these two groups. However, these results are consistent with WHO reports establishing that children and pregnant women are the most affected by soil-transmitted helminth infections [106].

All regions of Guinea presented prevalences of intestinal parasitic infections of more than 40% without significant differences. However, Upper Guinea was the region with the highest prevalence of *A. duodenale,* tapeworm, Trichine, and *H. nana.* Consistently, this region is considered with the highest degree of poverty [107]. On the other hand, in Upper Guinea, Forest Guinea, and Conakry, a high prevalence (all parasitosis combined) was noticed, but a significant decrease in prevalence during P3 was observed in all these localities.

## 7. Strengths and Limitations of the Study

A key strength of this systematic review and meta-analysis is that is the first to our knowledge to determine the pooled prevalence estimates of intestinal parasitic infections in Guinea. In addition, a rigorous search of several databases and other sources to identify eligible studies can also be considered one of the strengths of this review. On the other hand, one of the main limitations relates to a selection bias due to the fact that sampling in the majority of the studies was performed in healthcare structures since data from the general population in Guinea were limited and not available. The heterogeneity of the studies was also a limitation of the research.

Furthermore, concerning diagnostic tests, microscopic examination of fresh stools was the most commonly used method. In particular, techniques more adequate for the diagnosis of *E. vermicularis* and *S. stercoralis*, such as the Scotch tape test [100] and the Baermann [108], respectively, were not performed. Then, it is possible that some parasites were not routinely detected in clinical laboratories. WHO has recommended the Kato–Katz method as the best and most reliable diagnostic tool for the detection of human soil-transmitted helminths [109]. PCR would have been more sensitive in the identification and confirmation of several protozoans such as *Cryptosporidium* [110]. In addition, in all studies, only single stool sample tests were performed despite the recommendations suggesting at least three tests for the standard diagnosis [111]. Therefore, the prevalence was probably underestimated.

There was no adequate information on the distribution of prevalence according to sex, so it was not possible to evaluate whether the prevalence of intestinal parasitosis differed according to sex.

While the risk factors associated with pathogens could not be determined from the information provided in the selected studies, we suggest that they are likely to be related, in part, to factors such as socioeconomic status, access to potable water, and sanitation solutions. These factors were not explicitly described in many studies but are known as important predictors of intestinal parasitosis incidence in developing countries [10].

## 8. Conclusions

This is the first study in Guinea to assess the prevalence of intestinal parasitic infections in different regions of the country. It provides important data from 1989 until now, which may help in the conception and execution of public policies. We showed that intestinal parasitosis are a real health problem in Guinea, hence the need to put in place national strategies for regular control with a view to their eradication.

Prevention by deworming has been one of the most frequently applied strategies to fight against intestinal parasitosis. According to the WHO, large-scale preventive chemotherapy programs are required when the prevalence of any soil-transmitted helminth infection is higher than 20%, and this prevention is recommended twice a year when the baseline prevalence is over 50% [112]. Even if this strategy is one of the pillars of Guinea’s national NTD program, deworming is administered in this country only once a year. Moreover, this problem also requires actions based on more effective long-term solutions such as supplying better sanitation, access to clean drinking water, urban cleaning, solid waste management, and provision of improved drainage and management of urban rainwater [10]. In the near future, further research in the following areas is needed: outcomes of preventive chemotherapy; identification of households at risk; the correlation between the intensity of infection and morbidity; effects of co-interventions such as nutritional, environmental, water, hygiene, or sanitation; compliance with large-scale preventive chemotherapy programs, and effects of health education.

## Figures and Tables

**Table 1:** Description of different periods according to the National Program to Control Neglected Tropical Diseases.

	Target Population	Prevention Deworming Program	Frequency of Preventive Deworming	Administered Drugs (Doses)
Before 2010	No	No specific program	NA	Prazinquatel (600 mg), Mebendazol (500 mg)
2010–2013	Children between 5 and 12 years old assisting school and adults	One-time programs	Not periodically	Prazinquatel (600 mg), Albendazole (400 mg) or Mebendazol (500 mg)
After 2014	Populations aged 5 years and older	Mass treatment integrated in campaigns for lymphatic filariasis and onchocerciasis	Once a year	Prazinquatel (600 mg), Albendazole (400 mg) or Mebendazol (500 mg)

NA: non-applicable; mg: milligram. Data from [16].

**Table 2:** Characteristics of the selected studies included in the systematic review and meta-analysis.

Rank	Study Population Type (Age if Children)	Year of Observation	Study Area	Study Setting	Nb Included (Prevalence %)	Type of Parasite (Number of Cases)	Diagnostic Technique	References
1	Adult and children (1–17 years)	2006	Conakry (Urban)	Hospital	800 (23)	*S. stercoralis* (4), *A. duodenale* (42), *A. lumbricoides* (40), *E. histolytica* (27), *E. vermicularis* (3), *S. mansoni* (6), Tapeworm (11), Trichine (32), *T. intestinalis* (21)	A, D	Touré N., 2010, PharmD [20]
2	Children (1–17 years)	2008–2009	Conakry (Urban)	School	306 (43)	*S. stercoralis* (11), *A. duodenale* (24), *A. lumbricoides* (37), *E. histolytica* (21), *E. vermicularis* (3), *S. mansoni* (1), Tapeworm (6), Trichine (20), *T. intestinalis* (9)	A, D	Diallo G., 2010, PharmD [21]
3	Adult and children (1–17 years)	2009	Conakry (Urban)	Hospital	420 (42)	*S. stercoralis* (11), *A. duodenale* (41), *A. lumbricoides* (102), *S. mansoni* (8), Tapeworm (6), *T. trichiura* (7)	A, D	Assoumani M., 2010, PharmD [22]
4	Children (6–14 years)	2010	Lower Guinea (Kindia, Urban)	Community	612 (66)	*S. stercoralis* (9), *A. duodenale* (89), *A. lumbricoides* (113), *E. histolytica* (22), *E. vermicularis* (2), *S. mansoni* (86), Tapeworm (20), *T. trichiura* (12), *T. intestinalis* (48)	A, D	Camara A.B., 2010, PharmD [23]
5	Children (0–5 years)	2007	Conakry (Urban)	School	254 (80)	*S. stercoralis* (1), *A. duodenale* (23), *A. lumbricoides* (127), *E. histolytica* (7), *S. mansoni* (9), *T. trichiura* (26), *T. intestinalis* (9)	A, D	Diallo S.M.C., 2011, PharmD [24]
6	Children (5–17 years)	2008	Conakry (Urban)	School	300 (46)	*S. stercoralis* (2), *A. duodenale* (5), *A. lumbricoides* (104), *E. histolytica* (4), *H. nana* (3), *S. mansoni* (5), Tapeworm (3), *T. trichiura* (2), *T. intestinalis* (10)	A, D	Kolié F., 2011, PharmD [25]
7	Adults and children (1–17 years)	2008	Conakry (Urban)	Hospital	703 (87)	*S. stercoralis* (13), *A. duodenale* (73), *A. lumbricoides* (207), *E. histolytica* (105), *E. vermicularis* (58), Tapeworm (62), *T. intestinalis* (92)	A, D	Koné M.C., 2011, PharmD [26]
8	Adults and children (1–17 years)	2009	Conakry (Urban)	Hospital	300 (37)	*S. stercoralis* (5), *A. duodenale* (6), *A. lumbricoides* (32), *E. histolytica* (21), *E. vermicularis* (5), *H. nana* (5), *S. mansoni* (7), Tapewrom (26), *T. trichiura*(4)	A, D	Diallo A.A., 2011, PharmD [27]
9	Adults and children (1–17 years)	2009	Conakry (Urban)	Hospital	716 (47)	*S. stercoralis* (6), *A. duodenale* (8), *A. lumbricoides* (218), *E. histolytica* (58), *E. vermicularis* (1), *S. mansoni* (5), Tapeworm (39), *T. trichiura* (1), *T. intestinalis* (1)	A, D	Simpogui F.T., 2011, PharmD [28]
10	Children (2–17 years)	2009	Conakry (Urban)	Orphanat	99 (43)	*A. lumbricoides* (38), *S. mansoni* (1), Tapeworm (4)	A, D	Tidjani A., 2011, PharmD [29]
11	Children (9–14 years)	2010	Forest Guinea (Beyla and Macenta, Mixed)	School or community	420 (80)	*A. duodenale* (101), *A. lumbricoides* (34), *S. mansoni* (278), *T. trichiura* (10)	A, C	Hodges et al., 2011 [30]
12	Pregnant women	2011	Conakry (Urban)	Hospital	186 (47)	*A. duodenale* (10), *A. lumbricoides* (3), *E. histolytica* (4), *E. vermicularis* (5), *S. mansoni* (48), Tapeworm (8), *T. trichiura* (6), *T. intestinalis* (3)	A, D	Bilivogui F.G., 2011, PharmD [31]
13	Adults and children (1–17 years)	2010	Conakry and Lower Guinea (Kindia, Urban)	Community	4717 (42)	*S. stercoralis* (87), *A. duodenale* (556), *A. lumbricoides* (519), *E. vermicularis* (26), *S. mansoni* (242), Tapeworm (242), *T. trichiura* (303)	A, D	Barry A.R., 2012, PharmD [32]
14	Adults and children (0–15 years)	2010	Forest Guinea (N’Zérékoré, Urban)	Hospital	452 (39)	*S. stercoralis* (12), *S. mansoni* (165)	A, C	Kolié L., 2012, PharmD [33]
15	Pregnant women	2011	Conakry (Urban)	Hospital	121 (59)	*S. stercoralis* (12), *A. duodenale* (5), *A. lumbricoides* (29), *E. histolytica* (11), *S. mansoni* (3), Tapeworm (5), *T. intestinalis* (6)	A, D	Hounsanou E.L.D., 2012, PharmD [34]
16	Children (0–5 years)	2012	Middle Guinea (Mamou, Mixed)	Hospital	208 (46)	*S. stercoralis* (15), *A. lumbricoides* (41), *E. histolytica* (1), *E. vermicularis* (8), *G. intestinalis* (17), *S. mansoni* (1), *T. trichiura* (7), *T. intestinalis* (6)	X	Camara A.M., 2012, MD [35]
17	Adult and children (1–17 years)	2012	Forest Guinea (Macenta, Mixed	Community	515 (100)	*A. duodenale* (156), *A. lumbricoides* (124), *E. vermicularis* (12), *S. mansoni* (203), Tapeworm (10), Trichine (10)	A, D	Bilivogui A., 2013, PharmD [36]
18	Adults	2012	Conakry (Urban)	University	117 (68)	*A. duodenale* (1), *A. lumbricoides* (66), *E. vermicularis* (1), *H. nana* (1), Tapeworm (10)	A, D	Béavogui O.K., 2013, PharmD [37]
19	Pregnant women (15–17 years)	2012	Conakry (Urban)	Hospital	1975 (50)	*S. stercoralis* (139), *A. duodenale* (159), *A. lumbricoides* (199), *E. histolytica* (70), *E. vermicularis* (90), *S. mansoni* (50), Tapeworm (109), *T. trichiura* (179)	X	Condé M., 2013, PharmD [38]
20	Adults and children (0–17 years)	2012	Conakry (Urban)	Community	714 (53)	*S. stercoralis* (6), *A. duodenale* (35), *A. lumbricoides* (180), *E. vermicularis* (10), *F. buski* (50), *H. nana* (30), *T. trichiura* (70)	A, D	Kéita D., 2013, PharmD [39]
21	Children (1–17 years)	2012	Forest Guinea (Beyla, Mixed)	Community	779 (46)	*A. duodenale* (53), *A. lumbricoides* (24), *S. mansoni* (273), Tapeworm (7), Trichine (2)	A, D	Pricemou P., 2013, PharmD [40]
22	Pregnant women	2012	Conakry (Conakry, Urban)	Hospital	1488 (66)	*S. stercoralis* (93), *A. duodenale* (103), *A. lumbricoides* (165), *E. histolytica* (203), *E. vermicularis* (133), *S. mansoni* (145), Tapeworm (57), *T. intestinalis* (83)	A, B, D	Sow S., 2013, PharmD [41]
23	Adults and children (1–17 years)	2012	Conakry (Urban)	Hospital	3705 (27)	Helminths (1000)	X	Touré F., 2013, PharmD [42]
24	Children (1–17 years)	1989	Upper Guinea (Siguiri, Mixed	Community	1532 (70)	*S. stercoralis* (66), *A. duodenale* (298), *A. lumbricoides* (200), *E. histolytica* (65), *G. intestinalis* 6), *H. nana* (62), *S. mansoni* (119), Tapeworm (147), Trichine (113)	A, C, D	Diaby K., 2014, PharmD [43]
25	Adults and children (1–17 years)	2012	Forest Guinea (Macenta, Mixed)	Community	425 (52)	*A. duodenale* (74), *A. lumbricoides* (53), *E. histolytica* (11), *E. vermicularis* (8), *S. mansoni* (66), Tapeworm (5), Trichine (2)	A, D	Guilavogui D., 2014, PharmD [44]
26	Malnourished children (0–5 years)	2013	Forest Guinea (N’Zérékoré, Urban)	Hospital	62 (74)	*S. stercoralis* (1), *A. duodenale* (9), *A. lumbricoides* (15), *E. histolytica* (5), *E. vermicularis* (4), *G. intestinalis* (4), *S. mansoni* (5), Tapeworm (3)	A, D	Koïvogui F., 2014, MD [45]
27	Children (0–5 years)	2013	Forest Guinea (N’Zérékoré, Urban)	Hospital	400 (51)	*A. duodenale* (60), *A. lumbricoides* (66), *S. mansoni* (39), Tapeworm (6), *T. trichiura* (12), *T. intestinalis* (22)	A, D	Lamah L.S., 2014, PharmD [46]
28	Children (1–17 years)	2013	Conakry (Urban)	School	200 (34)	*A. duodenale* (14), *A. lumbricoides* (18), *H. nana* (10), *T. trichiura* (6), *T. intestinalis* (20)	A, D	Sano F., 2015, PharmD [47]
29	Pregnant women (1–17 years)	2014	Upper Guinea (Siguiri, Mixed)	Hospital	975 (70)	*S. stercoralis* (110), *A. duodenale* (128), *A. lumbricoides* (163), *E. histolytica* (52), *E. vermicularis* (87), *S. mansoni* (43), Tapeworm (99)	X	Camara M.S., 2015, PharmD [48]
30	Pregnant women	2014	Forest Guinea (N’Zérékoré, Mixed)	Hospital	780 (100)	*S. stercoralis* (126), *A. duodenale* (144), *A. lumbricoides* (156), *S. mansoni* (179), Tapeworm (71), *T. trichiura* (96), *T. intestinalis* (8)	A, D	Diabaté M., 2015, PharmD [49]
31	Adults and children VIH and TB (16–17 years)	2014	Conakry (Urban)	Hospital	123 (76)	*S. stercoralis* (1), *A. duodenale* (8), *A. lumbricoides* (44), *Cryptosporidium* (34), *E. vermicularis* (3), *G. intestinalis* (1), *H. nana* (1), *I. belli* (1), *S. mansoni* (1)	A, D, E	Gbamou R.N., 2015, PharmD [50]
32	Pregnant women (13–17 years)	2014	Lower Guinea (Kindia, Urban)	Hospital	200 (50)	*S. stercoralis* (10), *A. duodenale* (13), *A. lumbricoides* (36), *E. histolytica* (10), *S. mansoni* (9), Tapeworm (10), *T. trichiura* (12)	A	Hamza S.A., 2015, MSc [51]
33	Children (3–17 years)	2014	Conakry (Urban)	Hospital	780 (82)	*S. stercoralis* (7), *A. duodenale* (13), *A. lumbricoides* (390), *E. vermicularis* (7), *H. nana* (52), *S. mansoni* (20), Tapeworm (13), *T. trichiura* (138)	A, F	Moutsinga M.L.A., 2015, MSc [52]
34	Children (0–5 years)	2014	Forest Guinea (Macenta, Mixed)	Hospital	300 (41)	*S. stercoralis* (3), *A. duodenale* (22), *A. lumbricoides* (7), *E. histolytica* (32), *S. mansoni* (10), Tapeworm (23), *T. trichiura* (11), *T. intestinalis* (15)	A, B	Zoumanigui W., 2015, PharmD [53]
35	Children (1–17 years)	2015	Middle Guinea (Dalaba, Mixed)	School	430 (50)	*S. stercoralis* (19), *A. duodenale* (5), *A. lumbricoides* (34), *S. mansoni* (69), Tapeworm (84), *T. trichiura* 2)	A, D	Bah M.S., 2016, PharmD [54]
36	Children (0–15 years)	2015	Forest Guinea (Guéckedou, Mixed)	Hospital	820 (31)	*A. duodenale* (26), *A. lumbricoides* (156), *S. mansoni* (64), Tapeworm (4)	X	Sylla I.M., 2016, MD [55]
37	Adults and children (1–17 years)	2015–2016	Conakry (Urban)	Hospital	407 (76)	*S. stercoralis* (53), *A. duodenale* (40), *A. lumbricoides* (62), *S. mansoni* (47), Tapeworm (62), *T. trichiura* (47)	A	Camara A.K., 2016, MSc [56]
38	Children (5–15 years)	2017	Middle Guinea (Labé, Urban)	Hospital	372 (55)	*S. stercoralis* (5), *A. lumbricoides* (112), *S. mansoni* (11), Tapeworm (75)	A, B, D	DialloT.B., 2016, PharmD [57]
39	Adults and children (1–17 years)	2016	Middle Guinea (Labé, Urban)	Hospital	454 (41)	*A. duodenale* (12), *A. lumbricoides* (105), *E. histolytica* (6), *E. vermicularis* (4), *S. mansoni* (10), Tapeworm (46), *T. trichiura* (3)	A	Baldé A.S., 2017, MSc [58]
40	Pregnant women	2016	Conakry (Urban)	Hospital	384 (26)	*S. stercoralis* (2), *A. duodenale* (11), *A. lumbricoides* (59), *B. coli* (1), *E. histolytica* (1), *S. mansoni* (7), Tapeworm (15), Trichine (3), *T. intestinalis* (1)	A, D	Fotio C.S., 2017, PharmD [59]
41	Children (1–17 years)	2016	Conakry (Urban)	School	322 (43)	*S. stercoralis* (3), *A. duodenale* (5), *A. lumbricoides* (62), *E. vermicularis* (19), *S. mansoni* (7), Tapeworm (22), *T. trichiura* (21)	A, D	Kouyaté M., 2017, PharmD [60]
42	Children (0–15 years)	2016	Forest Guinea (N’Zérékoré, Mixed)	Hospital	504 (16)	Helminths (79)	X	Loua C., 2017, PharmD [61]
43	Children (0–5 years)	2017	Conakry (Urban)	Hospital	211 (27)	*A. duodenale* (6), *A. lumbricoides* (25), *E. histolytica* (9), *S. mansoni* (1), Tapeworm (11), *T. intestinalis* (5)	A, D	Kengni M.O., 2017, PharmD [62]
44	Adults and children (1–17 years)	2017	Conakry (Urban)	Hospital	354 (11)	*S. stercoralis* (2), *A. lumbricoides* (7), *E. histolytica* (7), *E. vermicularis* (1), *S. mansoni* (11), Tapeworm (9), *T. trichiura* (1)	A, B, D	Loua B., 2017, PharmD [63]
45	Children (0–5 years)	2017	Forest Guinea (Lola, Urban)	Hospital	160 (32)	*A. lumbricoides* (33), *E. histolytica* (15), Tapeworm (3)	A	Sagno I., 2017 MD [64]
46	Malnourished children (0–3 years)	2015	Conakry (Urban)	Hospital	220 (27)	*A. lumbricoides* (33), *B. coli* (11), *E. vermicularis* (13), Tapeworm (2)	A, D	Sidibé S et al., 2018 [3]
47	Pregnant women (16–42 years)	2016	Conakry (Urban)	Hospital	3571 (11)	*S. stercoralis* (27), *A. duodenale* (28), *A. lumbricoides* (91), *E. histolytica* (125), *E. vermicularis* (36), *S. mansoni* (29), Tapeworm (34), *T. trichiura* (23)	X	AbderamanI.B, 2018, PharmD [65]
48	Adults and children (1–17 years)	2016	Lower Guinea (Kindia, Urban)	Community	152 (34)	*S. mansoni* (52)	A, C	Bah O., 2018 Master [66]
49	Adults and children (1–17 years)	2016	Lower Guinea (Kindia, Mixed)	Community	477 (53)	*S. stercoralis* (5), *A. duodenale* (65), *A. lumbricoides* (181), *T. trichiura* (1)	A, C, D	Camara I.M., 2018, Master [67]
50	Human immunodeficiency virus (HIV) + adults and children (5–17 years)	2016	Conakry (Urban)	Hospital	38 (24)	*S. stercoralis* (1), *A. duodenale* (1), *E. histolytica* (3), *G. intestinalis* (1), *S. mansoni* (1), *T. intestinalis* (2)	X	Camara N., 2018, MD [68]
51	Children (3–5 years)	2016	Conakry (Urban)	Hospital	238 (29)	*S. stercoralis* (3), *A. duodenale* (4), *A. lumbricoides* (27), *E. histolytica* (13), *E. vermicularis* (10), *G. intestinalis* (6), Tapeworm (3), *T. trichiura* (3)	A, D	Guilavogui E., 2018, PharmD [69]
52	Children (0–15 years)	2016–2017	Forest Guinea (Kissidougou, Urban)	Hospital	265 (25)	*A. duodenale* (48), *E. histolytica* (9), *S. mansoni* (10)	A, F	Oularé M.L., 2018, MD [70]
53	Children (2–14 years)	2017	Conakry (Urban)	Hospital	384 (25)	*S. stercoralis* (5), *A. lumbricoides* (6), *H. diminuta* (13), *H. nana* (24), *S. mansoni* (15), Tapeworm (23), *T. trichiura* (11)	A, B, D	Ali I.O., 2018, PharmD [71]
54	Adults and children (1–17 years)	2017	Lower Guinea (Kamsar, Mixed)	Community	364 (42)	*S. stercoralis* (10), *A. duodenale* (19), *A. lumbricoides* (64), *E. vermicularis* (9), *S. mansoni* (11), Tapeworm (26), Trichine (12)	A, D	Bamba P., 2018, PharmD [72]
55	Children (0–5 years)	2017	Conakry (Urban)	Hospital	291 (25)	*A. lumbricoides* (36), *E. histolytica* (32), Tapeworm (4)	A	Camara O., 2018, MD [73]
56	Adults and children (5–17 years)	2017	Upper Guinea (Kankan, Urban=	Hospital	340 (86)	*S. stercoralis* (1), *A. duodenale* (91), *A. lumbricoides* (103), *F. s buski* (1), *S. mansoni* (88), Tapeworm (8)	A, C, D	Kolié O. 2018, PharmD [74]
57	Adults jailed	2017	Conakry (Urban)	Jail	330 (32)	*S. stercoralis* (7), *A. duodenale* (37), *A. lumbricoides* (10), *E. histolytica* (1), *S. mansoni* (47), Tapeworm (1), Trichine (2)	A, D	Kéita A., 2018, PharmD [75]
58	Adults and children (0–17 years)	2017	Forest Guinea (N’Zérékoré, Mixed)	Hospital	437 (41)	*S. stercoralis* (12), *A. duodenale* (56), *A. lumbricoides* (84), *E. histolytica* (2), Tapeworm (20), *T. trichiura* (6),	A, C	Onikoyamou K.H., 2018, MSc [76]
59	Children (0–5 years)	2017	Forest Guinea (Kissidougou, Urban)	Hospital	403 (18)	*A. duodenale* (12), *A. lumbricoides* (23), *E. histolytica* (7), *S. mansoni* (19), Tapeworm (11)	X	Mara F., 2018, MD [77]
60	Adults and children (2–17 years)	2017	Lower Guinea (Kamsar, Urban)	Hospital	300 (70)	*S. stercoralis* (1), *A. duodenale* (23), *A. lumbricoides* (82), *E. histolytica* (27), *E. vermicularis* (65), *S. mansoni* (1), Tapeworm (8), *T. trichiura* (3)	A	Traoré S., 2018, MSc [78]
61	Children (0–15 years)	2017–2018	Middle Guinea (Mamou, Mixed)	Hospital	590 (39)	*A. duodenale* (35), *A. lumbricoides* (129), *S. mansoni* (67)	A, C, D	Soumah A., 2019, MSc [79]
62	Adults and children living with HIV (16–17 years)	2014	Conakry (Urban)	Hospital	123 (82)	*S. stercoralis* (1), *A. duodenale* (8), *A. lumbricoides* (44), *Cryptosporidium* (33), *E. histolytica* (8) *E. vermicularis* (3), *G. intestinalis* (1), *H. nana* (1), *I. belli* (1), *S. mansoni* (2)	A, D, E	Toure D et al., 2019 [80]
63	Children (0–5 years)	2016	Upper Guinea (Kankan, Mixed)	Hospital	820 (15)	*A. lumbricoides* (73), *E. histolytica* (38), *G. intestinalis* (16)	A	Fofana L., 2019, MD [81]
64	Children (5–15 years)	2017	Lower Guinea (Coyah, Mixed)	Hospital	300 (19)	*S. stercoralis* (7), *A. duodenale* (27), *A. lumbricoides* (7), *H. nana* (2), *S. mansoni* (13), *T. trichiura* (2)	A, B, D	Gamou C.E., 2019, PharmD [82]
65	Children (0–14 years)	2018	Lower Guinea (Kindia, Urban)	Hospital	665 (38)	*A. duodenale* (37), *A. lumbricoides* (132), *E. vermicularis* (53), Tapeworm (29)	A, D	Diaby F., 2019, PharmD [83]
66	Adults and Children (1–17 years)	2018	Forest Guinea (N’Zérékoré, Mixed)	Community	2655 (53)	*S. stercoralis* (1), *A. duodenale* (10), *A. lumbricoides* (16), *E. histolytica* (3), *E. vermicularis* (1), Tapeworm (30), Trichine (3), *T. trichiura* (1), *T. intestinalis* (3), *S. mansoni* (1330)	A	Diallo M.S.M., 2019, Master [84]
67	Children (5–15 years)	2019	Conakry (Urban)	Hospital	315 (23)	*S. stercoralis* (1), *A. duodenale* (8), *A. lumbricoides* (37), *Balantidium coli* (1), *E. histolytica* (7) *S. mansoni* (3), Tapeworm (11), *T. intestinalis* (5)	A, C, D	Traoré S., 2019, PharmD [85]
68	Adults and children (6–12 years)	2017	Forest Guinea (Macenta, Urban)	Community	424 (100)	*S. stercoralis* (69), *A. duodenale* (26), *A. lumbricoides* (96), *S. mansoni* (24), Tapeworm (128), Trichine (81)	A, C, D	Béavogui S., 2020, PharmD [86]
69	Pregnant women	2019	Conakry (Urban)	Hospital	382 (76)	*S. stercoralis* (43), *A. duodenale* (81), *A. lumbricoides* (66), *E. histolytica* (40) *E. vermicularis* (41), Tapeworm (6), *T. trichiura* (15)	X	Condé A.F., 2020, PharmD [87]

*A. lumbricoides: Ascaris lumbricoides; A. duodenale: Ancylostoma duodenale; S. mansoni: Schistosoma mansoni; E. histolytica: Entamoeba histolytica; H. nana: Hymenolepis nana; E. vermicularis: Enterobius vermicularis; S. stercoralis: Strongyloides stercoralis; T. trichiura: Trichuris trichiura; Fasciolopis buski: Fasciolopsis buski; G. intestinalis: Giardia intestinalis; T. intestinalis: Trichomonas intestinalis; I. belli: Isospora belli; Balantidium coli: B. coli* Diagnostic technique: A: direct microscopic examination; B: formol-ether concentration technique; C: Kato–Katz technique; D: Willis flotation technique; E: modified Ziehl–Neelsen staining method; F: Coproculture; X: laboratory technique not described in the study. MD: medical doctor; MSc: master science; PharmaD: pharmacy doctor.

**Table 3:** Prevalence of different intestinal parasites in Guinea.

Parasites	N° of Studies	Sample Size	Cases	Prevalence (%) (95% CI) *	Heterogeneity	
					*p*-Value	I^2^ (%)
*A. lumbricoides*	61	38,279	5477	15 (12, 18)	0	98
*A. duodenale*	57	36,849	3114	6 (5, 8)	0	97
*S. mansoni*	55	35,256	3966	5 (3, 7)	0	99
*E. histolytica*	37	22,374	1044	4 (3, 5)	<0.01	93
Tapeworms	53	35,095	1677	3 (2, 4)	<0.01	96
*H. nana*	10	4573	188	3 (2, 5)	<0.01	73
*E. vermicularis*	34	26,965	816	2 (1, 3)	<0.01	95
*S. stercoralis*	46	31,785	1028	2 (1, 3)	<0.01	95
*T. trichiura*	32	24,193	1041	2 (1, 3)	<0.01	95
*G. intestinalis*	8	3144	52	2 (1, 4)	<0.01	86
*T. intestinalis*	20	10,977	369	2 (1, 4)	<0.01	91
Trichine	13	9068	280	1 (0, 3)	<0.01	95

* The prevalence of parasitosis were calculated by a random-effects meta-analysis. *A. lumbricoides: Ascaris lumbricoides; A. duodenale: Ancylostoma duodenale; S. mansoni: Schistosoma mansoni; E. histolytica: Entamoeba histolytica; H. nana: Hymenolepis nana; E. vermicularis: Enterobius vermicularis; S. stercoralis: Strongyloides stercoralis; T. trichiura: Trichuris trichiura; G. intestinalis: Giardia intestinalis; T. intestinalis: Trichomonas intestinalis.*

**Table 4:** Distribution of parasites according to regions.

			Prevalence * with 95% CI **			
	Conakry	Lower Guinea	Forest Guinea	Middle Guinea	Upper Guinea	*p* Value
*S. stercoralis*	1 (1, 2)	2 (1, 2)	1 (0, 6)	4 (2, 6)	1 (0, 1)	0.21
*A. duodenale*	4 (3, 5)	9 (6, 12)	9 (5, 17)	3 (1, 6)	22 (18, 28)	<0.01 ***
*A. lumbricoides*	19 (14, 26)	17 (9, 31)	10 (5, 17)	19 (13, 28)	16 (8, 27)	<0.01 ***
*E. vermicularis*	1 (1, 3)	4 (1, 16)	1 (0, 6)	2 (1, 5)	5 (3, 6)	<0.01 ***
*S. mansoni*	1 (1, 2)	5 (1, 19)	15 (8, 27)	4 (1, 11)	15 (6, 31)	<0.01 ***
Tapeworms	3 (2, 4)	4 (3, 6)	2 (1, 5)	16 (11, 22)	5 (2, 13)	<0.01 ***
Trichine	0 (0, 13)	3 (2, 6)	1 (0, 5)	-	7 (6, 9)	<0.01 ***
*T. trichiura*	3 (1, 6)	1 (0, 2)	1 (0, 4)	1 (0, 3)	-	<0.01 ***
*H. nana*	3 (2, 6)	1 (0, 2)	-	-	4 (3, 5)	0.04 ***
*G. intestinalis*	2 (1, 3)	-	6 (2, 16)	8 (5, 13)	1 (0, 3)	<0.01 ***
*E. histolytica*	5 (3, 7)	6 (3, 11)	2 (1, 6)	1 (1, 2)	4 (3, 5)	<0.01 ***
*T. intestinalis*	3 (2, 6)	8 (6, 10)	1 (0, 11)	3 (1, 6)	-	<0.01 ***

* The prevalence of parasitosis were calculated by a random-effects meta-analysis; ** CI: confidence interval; *** significative *p*-value.

## Data Availability

Not applicable.

## Supplementary Materials

The following supporting information can be downloaded at: https://www.mdpi.com/article/10.3390/pathogens12020336/s1. File S1: R code used for the analysis and the database.
